# Developing Multivariable Normal Tissue Complication Probability Model to Predict the Incidence of Symptomatic Radiation Pneumonitis among Breast Cancer Patients

**DOI:** 10.1371/journal.pone.0131736

**Published:** 2015-07-06

**Authors:** Tsair-Fwu Lee, Pei-Ju Chao, Liyun Chang, Hui-Min Ting, Yu-Jie Huang

**Affiliations:** 1 Medical Physics and Informatics Laboratory of Electronics Engineering, National Kaohsiung University of Applied Sciences, Kaohsiung 80778, Taiwan, ROC; 2 Graduate Institute of Clinical Medicine, Kaohsiung Medical University, Kaohsiung 807, Taiwan, ROC; 3 Department of Radiation Oncology, Kaohsiung Chang Gung Memorial Hospital and Chang Gung University College of Medicine, Kaohsiung, 83305, Taiwan, ROC; 4 Department of Medical Imaging and Radiological Sciences, I-Shou University, Kaohsiung 82445, Taiwan, ROC; University of California Davis, UNITED STATES

## Abstract

**Purpose:**

Symptomatic radiation pneumonitis (SRP), which decreases quality of life (QoL), is the most common pulmonary complication in patients receiving breast irradiation. If it occurs, acute SRP usually develops 4–12 weeks after completion of radiotherapy and presents as a dry cough, dyspnea and low-grade fever. If the incidence of SRP is reduced, not only the QoL but also the compliance of breast cancer patients may be improved. Therefore, we investigated the incidence SRP in breast cancer patients after hybrid intensity modulated radiotherapy (IMRT) to find the risk factors, which may have important effects on the risk of radiation-induced complications.

**Methods:**

In total, 93 patients with breast cancer were evaluated. The final endpoint for acute SRP was defined as those who had density changes together with symptoms, as measured using computed tomography. The risk factors for a multivariate normal tissue complication probability model of SRP were determined using the least absolute shrinkage and selection operator (LASSO) technique.

**Results:**

Five risk factors were selected using LASSO: the percentage of the ipsilateral lung volume that received more than 20-Gy (IV20), energy, age, body mass index (BMI) and T stage. Positive associations were demonstrated among the incidence of SRP, IV20, and patient age. Energy, BMI and T stage showed a negative association with the incidence of SRP. Our analyses indicate that the risk of SPR following hybrid IMRT in elderly or low-BMI breast cancer patients is increased once the percentage of the ipsilateral lung volume receiving more than 20-Gy is controlled below a limitation.

**Conclusions:**

We suggest to define a dose-volume percentage constraint of IV20< 37% (or AIV20< 310cc) for the irradiated ipsilateral lung in radiation therapy treatment planning to maintain the incidence of SPR below 20%, and pay attention to the sequelae especially in elderly or low-BMI breast cancer patients. (AIV20: the absolute ipsilateral lung volume that received more than 20 Gy (cc).

## Introduction

Radiation therapy is the most effective adjuvant treatment in breast cancer after surgery [1–3]. Lungs, located beneath the breasts, are among the most critical organs in radiation therapy in the treatment planning for breast cancer. As the essential organ for respiration, reduction of lung damage during breast cancer radiotherapy is important. Radiation pneumonitis (RP), which decreases quality of life (QoL), is the most common pulmonary complication in patients receiving breast irradiation. If it occurs, acute RP usually develops 4–12 weeks after completion of radiotherapy and presents symptoms as a dry cough, dyspnea and low-grade fever [4, 5]. If the incidence of symptomatic RP (SRP) is reduced, not only the QoL but also the compliance of breast cancer patients may be improved.

Several researchers have investigated the role of dosimetric predictive factors for RP in three-dimensional radiation therapy treatment planning, such as the percentage of the total lung volume exceeding a defined dose (Vdose) and ipsilateral mean lung dose (MLD), to reduce RP and/or escalate the radiation dose. Hernando *et al*. [6], Ramella *et al*. [7] and Graham *et al*. [8] have associated the percentage of lung volume receiving more than 20 Gy (V20), percentage of lung volume receiving more than 30 Gy (V30) and MLD with the risk of developing RP [7]. With the progression of radiation therapy techniques, intensity-modulated radiation therapy (IMRT) and hybrid IMRT used concurrently with conventional and IMRT beams create the most conformal dose distribution. However, more normal tissue receiving a low dose has been considered [9–11]. Additionally, the incidence of RP may depend on patient-related variables [12]. The risk factors for RP particularly with symptoms after radiation therapy using these novel technologies in breast cancer patients should be reviewed and analyzed more extensively.

Conventionally, the normal tissue complication probability (NTCP) model can be fitted using either univariate or multivariate logistic regression analysis to predict the incidence of SRP. However, the development of SRP likely depends on a variety of risk factors. Several variables such as clinical and patient-related factors, which may have important effects on the risk of radiation-induced complications, need to be considered. Therefore, evaluating the dose distribution of the lung volume involved as well as other potential clinical and treatment-related risk factors is important to develop predictive models.

The optimal number of risk factors for the model developed is controversial when the multivariate logistic regression model is used. Recently, Xu *et al*. [13, 14] and Lee *et al*. [15–17] recommended the least absolute shrinkage and selection operator (LASSO) method for multivariate logistic regression NTCP modeling. The LASSO method is based on shrinkage estimates and is widely used in the field of statistics. The advantages of LASSO include a smaller mean squared error (MSE), handling of the multicollinearity problem, overall variable selection and coefficient shrinkage [17, 18] and ease of implementation [19–23]. Therefore, in this study we developed a multivariate logistic NTCP regression model with LASSO to make valid predictions regarding the incidence of RP particularly with symptoms among breast cancer patients treated with hybrid IMRT.

## Methods

### Study population

Breast cancer patients referred to our department for adjuvant radiation therapy between April 2012 and November 2014 were evaluated, and those treated with hybrid IMRT were enrolled in this study. The patients’ characteristics, including basic information, disease status, and radiation therapy dosimetric parameters, were reviewed. This study was approved by Chang Gung Memorial Hospital institutional review boards of the hospital (103-0217B), and all participants provided written informed consent for the inclusion of their data in this retrospective analysis. All experiments were performed in accordance with relevant guidelines and regulations.

### Treatment techniques

The treatment plans were designed using the Philips Pinnacle^3^ treatment planning system (version 9.2, Philips, Fitchburg, WI, USA). Treatment was delivered by the computer-controlled auto-sequencing segment or the dynamic multileaf collimator of a linear accelerator [Varian Clinac 21 EX (Varian Medical Systems, Palo Alto, CA, USA) or Elekta Precise (Elekta, Crawley, UK)]. For each patient, treatment plans were implemented using the hybrid IMRT technique. The dose prescribed for the PTV was 50.4 Gy in 28 fractions. The optimization constraint ensured that the 95% isodose line encompassed 95% of the PTV (V95% ≥ 47.88 Gy).

The hybrid IMRT technique was combined with conventional and IMRT beams. The conventional conformal beams with medial and lateral beams designed for the tangential fields were used without wedges, and two anterior oblique IMRT beams were added [9–11]. The tangential beams were chosen so as to minimize exposure to the heart and lungs while ensuring adequate coverage of the PTV. The fields were extended 2–3 cm anteriorly of the breast to provide skin flashing of the region. The relative weights of the conventional beams were typically ≤ 83% of the dose delivered from conventional beams. The plans were optimized to cover the PTV and spare surrounding normal tissue (such as the lungs, heart and contralateral breast) as much as possible. All data were based on the dose-volume-histograms (DVHs) obtained using Pinnacle^3^ with a bin size resolution of 0.01 Gy. The resolution of the dose calculation was 2.5 mm for all IMRT plans.

### Evaluation of RP

The RP symptoms were evaluated during and after radiation therapy. Clinically symptomatic was defined according to the modified Common Toxicity Criteria of the National Cancer Institute of Canada (CTC-NCIC) [5, 24]: grade 0, no pneumonitis (no registered respiratory symptoms or respiratory problems independent of RT as determined by the oncologist); grade 1, mild pneumonitis (respiratory symptoms, i.e., cough and/or dyspnea with or without fever determined by the oncologist to be radiation-therapy-induced but not requiring steroids); grade 2, moderate complications (same as grade 1, but with impaired daily function and steroid treatment requirement). The most serious grading of the patient during and after radiation therapy was specified as the symptomatic severity.

Chest computed tomography (CT) was evaluated 1–3 months after completion of radiation therapy. Density changes on chest CT were evaluated by comparing with the CT image prior to radiation therapy for radiation therapy treatment planning. On both occasions, slices from three lung levels were examined (the central CT slice, the slice just above the heart contour and an apical slice at the clavicle level). An increase in density was graded according to a CT-adapted modification of Arriagada’s classification (0 = no change; 1 = low opacity in linear streaks; 2 = moderate opacity; 3 = complete opacity). The method used was introduced by Arriagada *et al*. and Lind *et al*., and the details can be found in the references[25, 26].

The final endpoint, acute SRP, was defined as patients with symptomatic pneumonitis combined with density changes ≥ grade 1 as measured on CT images.

### Statistical analysis

Data were fitted to a multivariate logistic NTCP regression model, and the method has been described in previous studies [15, 16, 27, 28]. For each patient, predictive values were calculated for each set of risk factors based on the multivariate logistic regression coefficients according to the following formula:

$$NTCP={\left(1+e^{-S}\right)}^{-1},\;\text{where}\;S=\beta_{0}+\sum_{i=1}^{n}\beta_{i}\cdot x_{i}$$ (1)

where *n* is the number of risk factors in the built model, variable *x*_*i*_ represents the different risk factors and *β*_*i*_ is the corresponding regression coefficient.

For each patient, 31 candidate risk factors were included initially in the variable selection procedure of this study. The candidate factors included 12 clinical and 19 dosimetric factors. The clinical candidate factors were age, BMI, Lung (d), Total (d), tumor site, chemotherapy, radiation energy, irradiation of IMNs, irradiation of supraclavicular fossa (SCF), surgery method, T stage, and N stage. The dosimetric candidate factors were the mean dose administered to the ipsilateral lung (MLD), the percentage of the ipsilateral lung volume (%) that received doses of 5–50 Gy at selected steps (IV5 ~IV50) and the absolute ipsilateral lung volume (cc) that received doses of 5–50 Gy at selected steps (AIV5 ~AIV50). We used the LASSO method to select the optimal number of potential risk factors for the predictive NTCP model. LASSO was first proposed by Tibshirani in 1996, and details can be found in the reference [29]; it uses the following eq to diminish the coefficients and select the risk factors:

$$\arg\;\min_{\beta }\;{\left\|Y-X\beta \right\|}^{2}\;\text{subject to}\;\left\|\beta \right\|=\sum_{j=0}^{n}\left|\beta_{j}\right|\leq t$$ (2)

where *n* is the number of variables selected, and t represents the tuning parameters that control the degree of penalty that can be determined by cross-validation [14, 18]. The default cross-validation in SPSS was used to obtain the best risk factor subsets. The risk factors selected were used for the definite NTCP model of SRP. After selecting the risk factors with the optimal performance, odds ratios (ORs) and 95% confidence intervals (95% CIs) were calculated for the selected risk factors in the model. Model performance was described using different validation tools [15, 18]. The system performance was expressed using the AUC (area under the receiver operating characteristic curve), Brier score, Nagelkerke’s R^2^, χ^2^, and Hosmer-Lemeshow test [15, 18].

The most significant dose-volume predictive factor determined was considered to fit the univariate logistic NTCP model for SRP, a model with traditional techniques that can be used conveniently. The parameters for the univariate NTCP regression model were obtained. Statistical analyses were performed using SPSS version 19.0 (SPSS, Chicago, IL, USA).

## Results

**Fig 1:**
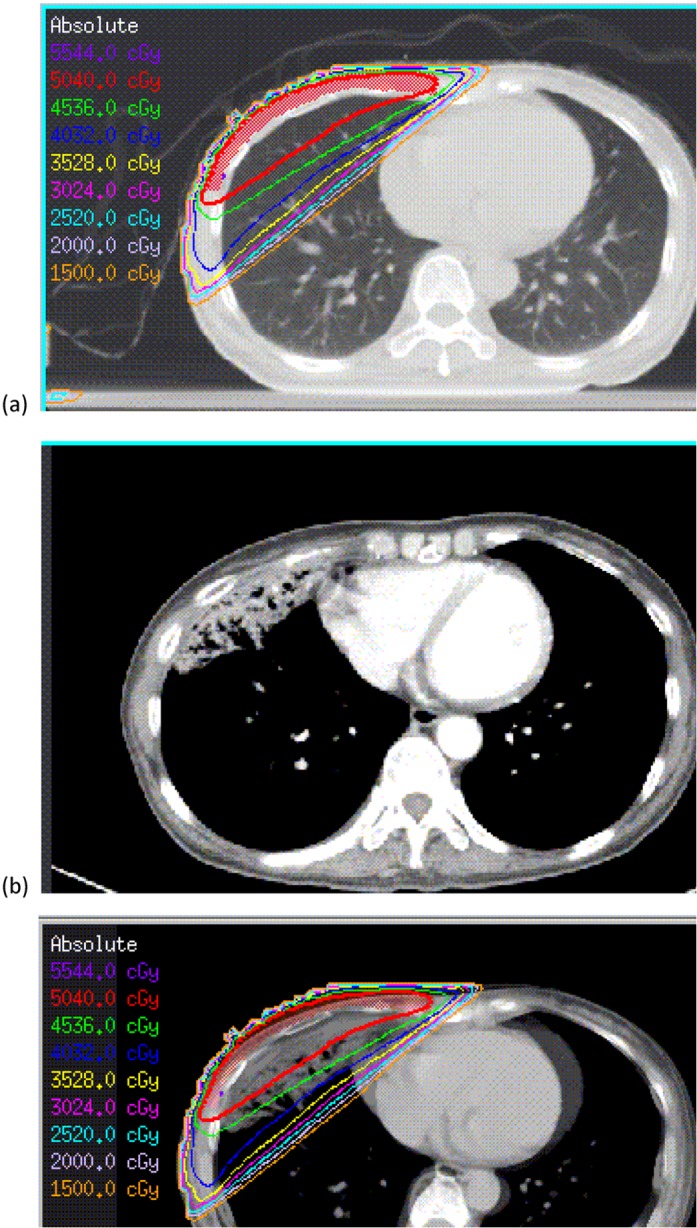
(a) A sample breast cancer treatment plan, (b) diagnosed with RP at 3 months after RT, (c) diagnosed with RP image fused with the original isodose curves. *Abbreviation*: RP: radiation pneumonitis; RT: radiotherapy.

In total, 93 patients were included in the analysis. After radiotherapy, 48 (52%), 29 (31%), 14 (15%) and 2 (2%) patients had lung density changes of grades 0, 1, 2 and 3, respectively, according to Arriagada’s classification and CT images [25, 26]. There were 50 patients (54%), 43 (46%) and 0 (0%) patients with clinical symptomatic pneumonitis of grades 0, 1 and 2, respectively, based on the CTC-NCIC [5, 24]. Patients without SPR were classified as group 0 (n = 62) and those with SPR as group 1 (n = 31). In total, 33.3% (31/93) of the patients suffered from SPR (Table 1). A typical breast cancer treatment plan is shown in Fig 1(A), an SRP diagnosis after RT is shown in Fig 1(B), and an image of diagnosed SRP fused with the original isodose curves is shown in Fig 1(C). The initial dosimetric and clinical predictive factors are shown in Table 2. The most significant dosimetric and clinical predictive factors for the logistic regression NTCP model were determined using the LASSO technique. First, factors were ranked based on how strongly they were correlated using LASSO; second, the optimal number of risk factors was chosen based on the Hosmer-Lemeshow test and the area under the receiver operating characteristic curve (AUC). The risk factors in the multivariate logistic regression analysis were ranked according to LASSO predictions in descending order as shown in Table 3.

**Table 1:** Characteristics of patients.

	Group (n = 93) Value—x (%)	Group 0 (n = 62) Value—x (%)	Group 1 (n = 31) Value—x (%)
**Age (y)**			
Median/Mean	55/54.6	55/53.5	55/56.7
Range	35–92	35–72	36–92
<51	26 (28)	21 (34)	5 (16)
51–60	42 (45)	26 (42)	16 (52)
61–70	22 (23)	14 (23)	8 (26)
>70	3 (4)	1 (1)	2 (6)
**BMI**			
Mean	24.6	25.1	23.7
Range	17.3–40.3	17.3–40.3	18.2–34.7
<21	12 (13)	4 (6)	8 (26)
21–26	53 (57)	38 (61)	15 (48)
>26	28 (30)	20 (33)	8 (26)
**Lung (d)**			
Mean	3.14	3.09	3.24
**Total (d)**			
Mean	6.03	6.14	5.81
**Tumor site**			
Left	44 (47)	32 (52)	12 (39)
Right	49 (53)	30 (48)	19 (61)
**SCF**			
NO	65 (70)	48 (77)	17 (55)
YES	28 (30)	14 (23)	14 (45)
**IMN**			
NO	79 (85)	53 (85)	26 (84)
YES	14 (15)	9 (15)	5 (16)
**Energy**			
6 MV	3 (3)	1 (2)	2 (6)
10 MV	13 (14)	11 (18)	2 (6)
6 MV+10 MV	77 (83)	50 (80)	27 (88)
**T stage**			
0	51 (55)	33 (53)	18 (58)
1	34 (37)	23 (37)	11 (35)
2	8 (8)	6 (10)	2 (7)
**N stage**			
0	47 (51)	31 (50)	16 (52)
1	27 (29)	20 (32)	7 (23)
2	19 (20)	11 (18)	8 (25)
**Surgery**			
PM	51 (55)	35 (57)	16 (52)
MRM	42 (45)	27 (43)	15 (48)
**Chemotherapy**			
NO	35 (38)	26 (42)	9 (29)
YES	58 (62)	36 (58)	22 (71)
**CT measured density changes**			
Grade 0	48 (52)	48	
Grade 1	29 (31)		29
Grade 2	14 (15)		14
Grade 3	2 (2)		2
**Symptomatic pneumonitis**			
Grade 0	50 (54)	50	
Grade 1	43 (46)		43
Grade 2	0		0
**SRP**			
Group 0	62 (67)	62	
Group 1	31 (33)		31

*Abbreviation*: Group 0: Without symptomatic radiation pneumonitis; Group 1: Symptomatic radiation pneumonitis

BMI: body mass index; SCF: Irradiation to supraclavicular fossa; IMN: Irradiation to internal mammary lymph nodes; PM: partial mastectomy; MPM: modified radical mastectomy; SRP: Symptomatic radiation pneumonitis; Lung (d): maximum Lung depth from chest wall to tangential line between extreme points of PTV in the section of nipple on CT image

Total (d): maximum depth from skin to tangential line between extreme points of PTV in the section of nipple on CT image

**Table 2:** Candidate predictive factors initially.

No.	Description	Range or Classification	Median or frequency	p-valve
1	MLD	13.76–28.64	20.52	0.005
2	IV5	24.43–83.56	60.41	0.014
3	IV10	32.20–68.05	49.64	0.005
4	IV13	18.22–62.73	45.74	0.003
5	IV15	26.84–60.49	44.21	0.003
6	IV20	23.28–56.61	40.99	0.001
7	IV25	20.52–53.79	37.88	0.004
8	IV30	17.99–50.74	35.31	0.008
9	IV40	10.37–44.67	28.29	0.038
10	IV50	2.49–32.41	11.37	0.188
11	AIV5	304.64–1214.54	711.23	0.053
12	AIV10	288.86–977.83	584.86	0.039
13	AIV13	227.20–908.85	539.45	0.022
14	AIV15	222.91–877.27	522.36	0.025
15	AIV20	187.53–818.15	483.85	0.017
16	AIV25	167.25–772.34	447.08	0.019
17	AIV30	152.15–730.36	417.27	0.033
18	AIV40	121.43–631.27	333.60	0.066
19	AIV50	16.14–393.78	133.45	0.118
20	Age	35–92	54.61	0.132
21	BMI	17.35–40.35	24.64	0.093
22	Lung (d)	0.81–5.23	3.14	0.368
23	Total (d)	2.98–10.13	6.03	0.345
24	Tumor site	0,1^#^	44,49	0.242
25	Chemotherapy	0,1*	35,58	0.229
26	Energy	0,1,2^&^	3,13,77	0.212
27	IMN	0,1*	79,14	0.838
28	SCF	0,1*	65,28	0.028
29	Surgery	0,1^ѱ^	51,42	0.659
30	T stage	0,1,2	51,34,8	0.840
31	N stage	0,1,2	47,27,19	0.517

*Abbreviation*: BMI: body mass index; SCF: Irradiated to supraclavicular fossa; IMN: Irradiated to internal mammary lymph nodes

MLD: mean dose to the ipsilateral lung; IV5-IV50: ipsilateral lung volume received 5-50Gy (%); p-valve: univariate logistic test; AIV5-AIV50: absolute ipsilateral lung volume received 5-50Gy (cc); p-valve: univariate logistic test

^#^0 = Left, 1 = Right

*0 = No, 1 = Yes

^ѱ^0 = PM (partial mastectomy), 1 = MRM (modified radical mastectomy)

^&^0 = 6MV, 1 = 10MV, 2 = 6+10MV; T stage: 0 = T0, T1 (T1a, T1b, T1c, T1m, T1s), 1 = T2, 2 = T3, T4 (T4a, T4b)

N stage: 0 = N0, 1 = N1 (N1, N1a), 2 = N2 (N2, N2a), N3 (N3, N3a, N3b)

Lung (d): maximum Lung depth from chest wall to tangential line between extreme points of PTV in the section of nipple on CT image; Total (d): maximum depth from skin to tangential line between extreme points of PTV in the section of nipple on CT image

**Table 3:** Predictive factors correlation ranked by LASSO.

Predictive factors							
1.	IV20	9.	Tumor site	17.	AIV25	25.	IV25
2.	Energy	10.	Lung (d)	18.	IV13	26.	AIV30
3.	Age	11.	IV40	19.	AIV50	27.	IV50
4.	BMI	12.	Surgery	20.	MLD	28.	IV15
5.	T stage	13.	AIV40	21.	AIV5	29.	AIV10
6.	SCF	14.	IV10	22.	IV5	30.	AIV15
7.	Chemotherapy	15.	IV30	23.	Total (d)	31.	IMN
8.	N stage	16.	AIV20	24.	AIV13		

*Abbreviation*: LASSO: least absolute shrinkage and selection operator; BMI: body mass index

SCF: Irradiated to supraclavicular fossa; IMN: Irradiated to internal mammary lymph nodes; MLD: mean dose to the ipsilateral lung; IV5-IV50: ipsilateral lung volume received 5-50Gy (%); AIV5-AIV50: absolute ipsilateral lung volume received 5-50Gy (cc); Definition of risk factors: Same as Table 2.

Lung (d): maximum Lung depth from chest wall to tangential line between extreme points of PTV in the section of nipple on CT image; Total (d): maximum depth from skin to tangential line between extreme points of PTV in the section of nipple on CT image

The optimal number of risk factors selected using LASSO with cross validation was five, which included the percentage of the ipsilateral lung volume that received more than 20 Gy (IV20), energy, age, body mass index (BMI) and T stage. All corresponding coefficients of the multivariate logistic regression NTCP models are shown in Table 4. The NTCP value for each individual patient can be calculated using the following logistic regression formula: *NTCP* = (1 + *e* ^−*S*^)^−1^, the optimal model, where

$$\begin{array}{l} \text{S}=\text{-}6.868+(\text{IV}20\;*0.183)+(\text{Energy}\;*\;\text{corresponding value})+(\text{Age}\;*0.045)+(\text{BMI}\;*\text{-}0.093)+(\text{T stage}\;*\\ \text{corresponding value}). \end{array}$$

**Table 4:** Multivariate logistic regression coefficients and odds ratios for the NTCP model.

Predictive factors (n = 5)	β	*p*	Odds Ratio	95% CI
IV20	0.183	0.001	1.201	1.083–1.330
Energy^&^ (6MV)		0.166		
E(1) (10MV)	-2.576	0.103	0.076	0.003–1.676
E(2) (6+10MV)	-1.164	0.401	0.312	0.021–4.712
Age	0.045	0.103	1.046	0.991–1.105
BMI	-0.093	0.207	0.911	0.788–1.053
T stage (T0, T1)		0.207		
T(1) (T2)	-0.904	0.139	0.405	0.122–1.343
T(2) (T3, T4)	-1.369	0.166	0.254	0.037–1.766
Constant	-6.868	0.041	0.001	

*Abbreviation*: IV20: the ipsilateral lung volumes receiving doses of 20Gy; BMI: body mass index; CI: confidence interval

^&^0 = 6MV, 1 = 10MV, 2 = 6+10MV; T stage: 0 = T0, T1 (T1a, T1b, T1c, T1m, T1s), 1 = T2, 2 = T3, T4 (T4a, T4b)

Statistical positive associations were observed among the incidence of SRP, IV20, and patient age. Energy, BMI and T stage showed a negative association with SRP incidence but without significance.

**Fig 2:**
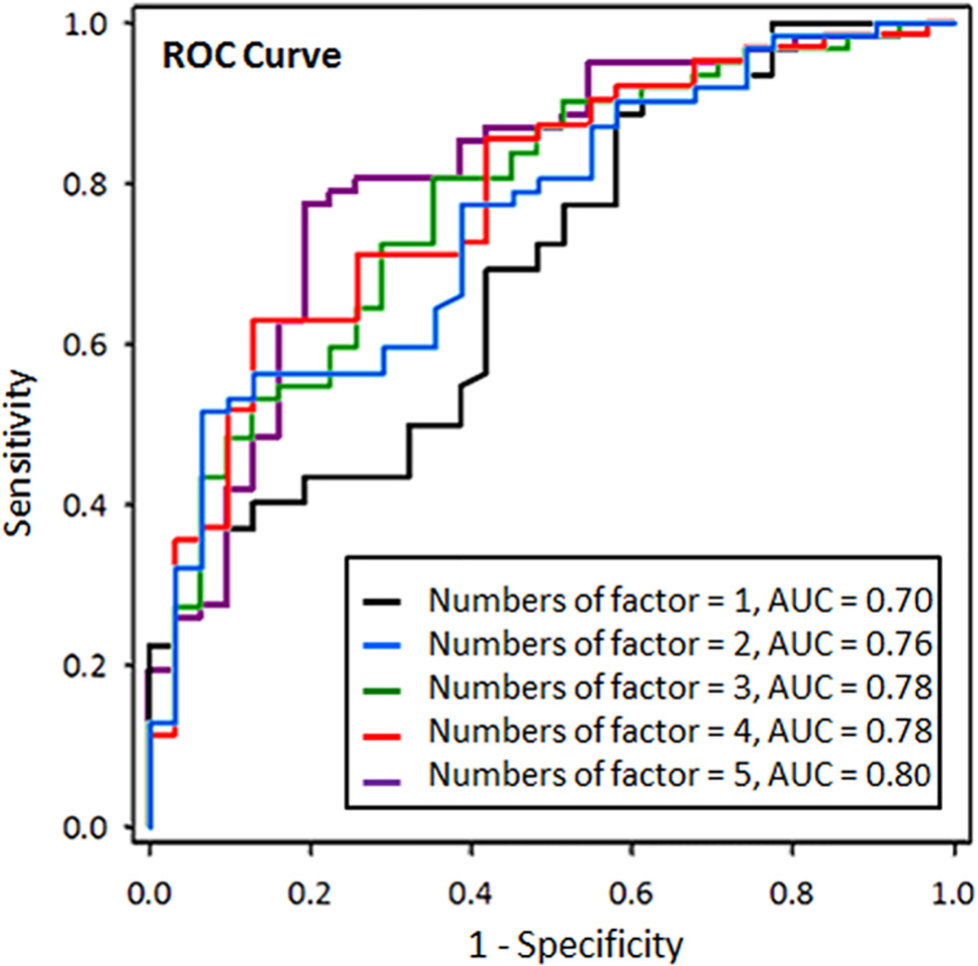
The receiver operating characteristic curves (ROC) of the five normal tissue complication probability models for symptomatic radiation pneumonitis in breast cancer patients treated with hybrid IMRT. Five factors: IV20, energy, age, body mass index (BMI) and T stage.

The overall performance of the NTCP model for SRP incidence in terms of the AUC, Nagelkerke R^2^, Omnibus, and Hosmer–Lemeshow test was satisfactory and corresponded well with the expected values. The AUC of the optimal model was 0.80 (95% CI 0.71–0.90). Finally, the Hosmer-Lemeshow test showed a significant correlation between predicted risk and observed outcome for the LASSO optimized model (Table 5). The performance of the NTCP model using one to five predictors is shown in Table 5. Comparison of the receiver operating characteristic curves of the five NTCP models for SRP were shown in Fig 2.

**Table 5:** System performance evaluation.

Number of factors	AUC (CI95%)	R^2^ Nagelkerke	Omnibus	Hosmer–Lemeshow
1	0.70 (0.58–0.80)	0.197	<0.001	0.228
2	0.76 (0.66–0.86)	0.250	<0.001	0.220
3	0.78 (0.68–0.88)	0.287	<0.001	0.613
4	0.78 (0.68–0.88)	0.304	<0.001	0.892
5	0.80 (0.71–0.90)	0.344	<0.001	0.201
6	0.80 (0.71–0.90)	0.352	<0.001	0.022

*Abbreviation*: AUC: Area under the receiver operating characteristic curve; HL: Hosmer–Lemeshow test; the first five predictive factors were IV20, energy, age, BMI, and T stage.

**Fig 3:**
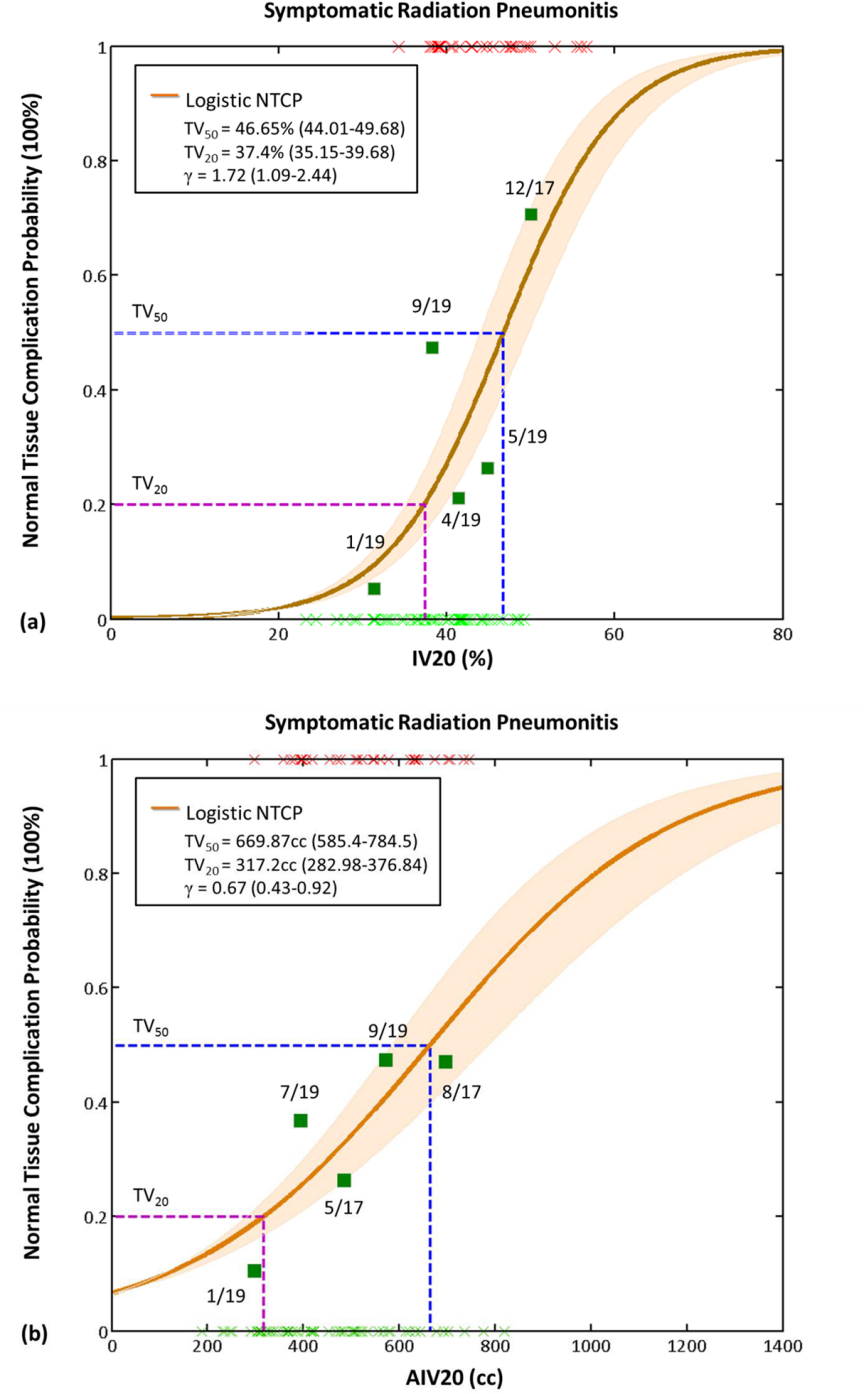
The univariate logistic normal tissue complication probability models with (a) IV20 and (b) AIV20 for symptomatic radiation pneumonitis in breast cancer patients treated with hybrid IMRT. *Abbreviation*: IV20: ipsilateral lung volume received >20Gy (%); AIV20: absolute ipsilateral lung volume received >20Gy (cc); IMRT: intensity modulated radiotherapy.

The univariate dose-response fitted curve (using IV20 and AIV20) for the incidence of SRP in breast cancer patients treated with hybrid IMRT is shown in Fig 3A and 3B. The parameters for the univariate NTCP regression analysis shown in Fig 3 were calculated using the percentage and the absolute of the ipsilateral lung volume that received more than 20 Gy (IV20 and AIV20). According to the NTCP curve, we determined the tolerances of IV20 and AIV20 producing a 50% complication rate (TV_50_) to be 46.7% and 660cc in breast cancer patients treated with hybrid IMRT, respectively. The tolerances IV20 and AIV20 corresponding to a 20% incidence of complications (TV_20_) was ≈ 37% and 310cc, respectively.

## Discussion

RP is the most common pulmonary complication in patients receiving breast irradiation [4, 5]. RP reflects the response to radiation, including loss of pneumocytes, increased capillary permeability, interstitial and alveolar edema, and access to inflammatory cell intra-alveolar spaces [30]. Changes are also observed in the radiographic features, especially on CT, which not only delineates parenchymal changes better but also demonstrates the changes restricted to the irradiated area. The most common findings are ground-glass opacities and/or airspace consolidation [31, 32]. In this study, although CT density changes were found in 45 patients (48.3%), 43 patients (46.2%) had complaints consistent with pneumonitis symptoms. Because the pneumonitis symptoms are not specific, differential diagnosis is difficult. Conversely, asymptomatic RP does not affect patients’ radiation therapy schedule or QoL. SRP should be more significant during radiation therapy in breast cancer patients. Therefore, establishing an NTCP model for symptomatic RP in radiation therapy for breast cancer is more important and meaningful.

Generally, the models developed in a population treated using a specific technique cannot be generalized and extrapolated to a population treated using another technique without external validation. Beetz *et al*. showed that 3D conformal radiation therapy (3D-CRT)-based models for patient-rated xerostomia among patients treated with 3D-CRT were less valid for patients treated with IMRT, and that the 3D-CRT NTCP models could not be used for IMRT cohorts [33]. Therefore, validation was performed for the breast cancer patients treated with hybrid IMRT instead of using the parameters obtained from 3D-CRT. Prediction of RP in breast cancer patients can be improved by using multivariate logistic regression models with the LASSO technique. When the number of factors used was increased from one (MLD) to five (IV20, energy, age, BMI and T stage), the AUC value improved from 0.67 to 0.80. Based on the associations identified, an efficient set of predictive factors can be determined to limit the risk of SRP in breast cancer patients treated with hybrid IMRT. The predictive factors included in the models are useful to optimize current IMRT treatments with regard to SRP and to indicate which predictive factors are the most important to minimize exposure of healthy tissue.

According the results of this study, the incidence of SRP in breast cancer patients after hybrid IMRT was 33.3%. The most significant risk factors selected were IV20, energy, age, BMI and T stage. Regarding dosimetric parameters, we obtained 19 absolute and percentage of ipsilateral lung volumes for V5 Gy to V50 Gy doses at specific intervals and the mean dose to the ipsilateral lung as the model predictor. The most significant dose-volume predictive factor was determined using the LASSO technique with IV20 for the logistic regression NTCP model. When only the dosimetric parameters were ranked, the orders were sorted as IV20, IV40, AIV40, IV10, IV30, AIV20, AIV25, IV13, AIV50, MLD, AIV5, IV5, AIV13, IV25, AIV30, IV50, IV15, AIV10, and AIV15. Ramella *et al*. [7] showed that adding IV20 and IV30 to the classical total lung constraints reduced pulmonary toxicity in concurrent chemoradiation treatment. They suggested that not all beam entrances should be on the ipsilateral lung. A conservative approach would be to use the constraint settings IV20 and IV30 as simple predictive factors of lung injury. This is consistent with our results showing that IV20 was the greatest risk predictor for the model; as IV20 increased, the RP incidence increased. Goldman *et al*. [34] showed that minimizing the percentage of the ipsilateral lung dose to V20 < 30% can significantly reduce moderate to severe radiological changes and symptomatic pneumonitis based on chest X-rays. In the current study, when the dose to the ipsilateral lung volume was limited to IV20 < 30%, the incidence of RP was 0% in our cohort treated with hybrid IMRT. Lind *et al*. [4] concluded that the incidence of short-term moderate pulmonary complications in adjuvant locoregional 3D RT for breast cancer is clinically significant. Their results also suggested an association of pulmonary complications with increasing age and incidentally irradiated lung volume (IV20). These results are in agreement with our analysis for the patients treated with hybrid IMRT. This is one advantage of transitioning into the era of hybrid IMRT and potentially more unconstrained beam arrangements [8].

In this study, a significant correlation between IV20 and MLD was found. The covariate MLD did not significantly influence the outcome based on multivariate analysis. The mean dose administered to the ipsilateral lung did not show significance in the group with grade 1+ RP toxicity. The MLDs were 21.76 Gy and 19.89 Gy for the groups with and without RP toxicity, respectively. Conversely, IV20 was the most significant dosimetric predictor for the model; therefore, a single IV20 univariate NTCP regression model was considered for convenience use. To our knowledge, no univariate NTCP model IV20 exists. In the univariate NTCP analysis, the TV_50_ for IV20 volume (50% cutoff point) was 46.7% in breast cancer patients treated with hybrid IMRT. We suggest that to maintain the incidence of grade SRP below 20%, the percentage IV20 volume should be limited to < 37%. For AIV20, TV_50_ for AIV20 volume was 660cc and TV_20_ for AIV20 volume was 310cc, respectively.

The constrains of lungs could be intervened and controlled well in a careful radiation therapy treatment planning. However, there are still innate patients’ characteristics for the risk of SPR. Once the dosimetric issue was controlled, patient age (≥ 55 years, median age) was associated with the risk of pulmonary complications. Our results are consistent with other studies on breast cancer patients [35–38]. Kahán *et al*. indicated that the risks of early and late radiogenic lung sequelae after 3D conformal radiotherapy in breast cancer patients were strongly associated with patient age, volume of the irradiated lung, and the applied dose [35]. Their results show that increased attention is necessary when radiotherapy is administered to patients over 59 years of age. Age is one of the most significant predictors of lung sequelae in patients treated using hybrid IMRT. Increasing age has been correlated with both clinical symptoms and radiological changes [4, 26].

Furthermore, BMI showed a negative, but not significant, association with the incidence of RP in patients treated with hybrid IMRT. Abe *et al*. [39] reported similar results; the BMIs of symptomatic patients were significantly lower than those of asymptomatic patients (*p* = 0.014). Hernando *et al*. [6] illustrated that relative weight loss during the 6-month period before RT referral was associated with fewer cases of RP, which contradicts our results. Allen *et al*. [40] retrospectively analyzed the records of 200 node-positive breast cancer patients treated with 3D-CRT. Of the 14 patients with RP, 9 (64%) had a BMI > 27, compared with 9 out of the 42 (21%) patients in the control group (*p* = 0.0065). Although BMI was correlated highly with RP, pulmonary comorbidities and IV20 were also relevant. Physicians should consider these risk factors when treating patients. However, treatment methods may differ among nations and institutions. Differences in area and radiation modality may produce different types and levels of RP toxicity. Energy and T stage showed a non-significant negative association with the incidence of RP in patients treated with hybrid IMRT. Using higher energy photons not only improve skin sparing effect but also can obtain better target dose uniformity and lacking lateral scattering in the low-density lung [41], making SRP less than the lower energy modality had. The treatment target after surgical intervention may be the cause of negative association between the T-stage and the incidence of SRP. Early stage breast cancer performed partial mastectomy (PM) mostly, whereas modified radical mastectomy (MRM) for advanced stage. The target after PM includes the whole beast and boost, which implies a higher and ununiformed dose distribution. The target after MRM is the chest wall with /without axilla. The shape of the target is smoother and easily to achieve a satisfied requirement in treatment planning. No matter how, there is no statistic significant in Energy and T stage.

Post-chemotherapy status, a non-dosimetric factor that may affect the risk of SRP in patients, is an issue that needs to be addressed. Our report showed no association between SRP and chemotherapy, which is in agreement with other reported data [42, 43]. Lind *et al*. [43] showed that pulmonary complications and concurrent tamoxifen intake or pre-RT adjuvant chemotherapy were not correlated. However, other treatment series have reported associations between chemotherapy and RT [25, 44].

In this study, the irradiation of internal mammary nodes (IMNs) was not statistically significant in the multivariate model. To irradiate IMNs, the irradiated field extends to the sternum-costal space, which may increase the radiation exposure to the lung. However, IMN irradiation was ranked thirty-first and did not significantly influence the outcome based on multivariate analysis. Hooning *et al*. [45] showed in cases of IMN irradiation, via a direct anterior field, that the heart and coronary arteries are exposed to similar doses in the left- and right-side fields. In their study, many patients received radiation to IMNs via the anterior field, and no difference in risk between left- and right-sided tumors was found. Lind *et al*. [4] reported that the frequency and severity of pulmonary complications in patients treated with locoregional RT including IMNs (approximately 10% treated with corticosteroids) created a clinically significant problem, especially when RT was used as an adjuvant. In the present study, the univariate analysis for IMNs showed no significance (*p* = 0.92). Similarly, the factors that may affect the irradiated volume of the lungs, Lung (d) and Total (d), were not significant for SRP in this study. The definition of Lung (d) is the maximum lung depth from the chest wall to the tangential line between extreme planning target volume (PTV) points in the nipple area on CT images, and Total (d) is the maximum depth from skin to tangential line between extreme PTV points in the nipple area on CT images. The dosimetric factors still appear to be the most significant for symptomatic pneumonitis based on the results of this study.

Smoking may be a significant predictor for the risk of pulmonary complications. Theuws *et al*. [38] and Johansson *et al*. [46] found a lower incidence of RP in breast cancer patients who smoked. Kahán *et al*. [35] showed an association between smoking and radiation lung injury in breast cancer patients that is controversial. However, we did not take the effects of smoking into consideration because at the time of RT referral only three patients were smokers and none of these had SRP.

SRP not only reduces the QoL but also the compliance of breast cancer patients in radiation therapy. A careful radiation therapy treatment planning in breast irradiation could overmaster dosimetric issue to lung. However, innate patients’ characteristics are still to be the risks for SPR. Therefore, it should pay more attention to the elderly or low-BMI breast cancer patients before and after radiation therapy in clinical practice. On the other hand, this study was limited by the small number of patients available. The predictive power could be improved by increasing the sample size. In addition, the selected NTCP parameters could be generalized and extrapolated to other breast cancer patient groups or radiation therapy for other diseases in future studies.

## Conclusion

Our analyses indicate an increased risk of SRP in elderly and low-BMI breast cancer patients once the percentage of ipsilateral lung volume that receives more than 20-Gy of hybrid IMRT is under controlled. The risk factors included in the model are useful to optimize current hybrid IMRT planning. We suggest to a dose-volume constraint of IV20 < 37% (or AIV20 < 310cc) for the irradiated ipsilateral lung to maintain incidence of SPR below 20%, and pay attention to the sequelae especially in elderly or low-BMI breast cancer patients.

## References

[1] Clarke M, Collins R, Darby S, Davies C, Elphinstone P, Evans E, et al Effects of radiotherapy and of differences in the extent of surgery for early breast cancer on local recurrence and 15-year survival: an overview of the randomised trials. Lancet. 2005;366(9503):2087–106. 10.1016/S0140-6736(05)67887-7 . 16360786

[2] Early Breast Cancer Trialists' Collaborative G, Darby S, McGale P, Correa C, Taylor C, Arriagada R, et al Effect of radiotherapy after breast-conserving surgery on 10-year recurrence and 15-year breast cancer death: meta-analysis of individual patient data for 10,801 women in 17 randomised trials. Lancet. 2011;378(9804):1707–16. 10.1016/S0140-6736(11)61629-2 22019144 PMC3254252

[3] Ebctcg, McGale P, Taylor C, Correa C, Cutter D, Duane F, et al Effect of radiotherapy after mastectomy and axillary surgery on 10-year recurrence and 20-year breast cancer mortality: meta-analysis of individual patient data for 8135 women in 22 randomised trials. Lancet. 2014;383(9935):2127–35. 10.1016/S0140-6736(14)60488-8 . 24656685 PMC5015598

[4] Lind PA, Wennberg B, Gagliardi G, Fornander T. Pulmonary complications following different radiotherapy techniques for breast cancer, and the association to irradiated lung volume and dose. Breast Cancer Res Treat. 2001;68(3):199–210. 11727957 10.1023/a:1012292019599

[5] Rancati T, Wennberg B, Lind P, Svane G, Gagliardi G. Early clinical and radiological pulmonary complications following breast cancer radiation therapy: NTCP fit with four different models. Radiother Oncol. 2007;82(3):308–16. 17224197 10.1016/j.radonc.2006.12.001

[6] Hernando ML, Marks LB, Bentel GC, Zhou S-M, Hollis D, Das SK, et al Radiation-induced pulmonary toxicity: a dose-volume histogram analysis in 201 patients with lung cancer. International journal of radiation oncology, biology, physics. 2001;51(3):650–9. 11597805 10.1016/s0360-3016(01)01685-6

[7] Ramella S, Trodella L, Mineo TC, Pompeo E, Stimato G, Gaudino D, et al Adding Ipsilateral V20 and V30 to Conventional Dosimetric Constraints Predicts Radiation Pneumonitis in Stage IIIA–B NSCLC Treated With Combined-Modality Therapy. International journal of radiation oncology, biology, physics. 2010;76(1):110–5. 10.1016/j.ijrobp.2009.01.036 19619955

[8] Graham MV, Purdy JA, Emami B, Harms W, Bosch W, Lockett MA, et al Clinical dose–volume histogram analysis for pneumonitis after 3D treatment for non-small cell lung cancer (NSCLC). International journal of radiation oncology, biology, physics. 1999;45(2):323–9. 10487552 10.1016/s0360-3016(99)00183-2

[9] Mansouri S, Naim A, Glaria L, Marsiglia H. Dosimetric evaluation of 3-D conformal and intensity-modulated radiotherapy for breast cancer after conservative surgery. Asian Pacific journal of cancer prevention: APJCP. 2014;15(11):4727–32. . 24969911 10.7314/apjcp.2014.15.11.4727

[10] Mayo CS, Urie MM, Fitzgerald TJ. Hybrid IMRT plans—concurrently treating conventional and IMRT beams for improved breast irradiation and reduced planning time. International journal of radiation oncology, biology, physics. 2005;61(3):922–32. 10.1016/j.ijrobp.2004.10.033 . 15708276

[11] Michalski A, Atyeo J, Cox J, Rinks M, Morgia M, Lamoury G. A dosimetric comparison of 3D-CRT, IMRT, and static tomotherapy with an SIB for large and small breast volumes. Medical dosimetry: official journal of the American Association of Medical Dosimetrists. 2014;39(2):163–8. 10.1016/j.meddos.2013.12.003 . 24393498

[12] Tsougos I, Mavroidis P, Rajala J, Theodorou K, Järvenpää R, Pitkänen MA, et al Evaluation of dose–response models and parameters predicting radiation induced pneumonitis using clinical data from breast cancer radiotherapy. Phys Med Biol. 2005;50(15):3535 16030381 10.1088/0031-9155/50/15/004

[13] van der Schaaf A, Xu C-J, van Luijk P, van’t Veld AA, Langendijk JA, Schilstra C. Multivariate modeling of complications with data driven variable selection: Guarding against overfitting and effects of data set size. Radiother Oncol. 2012;105(1):115–21. 10.1016/j.radonc.2011.12.006 22264894

[14] Xu C-J, van der Schaaf A, Van't Veld AA, Langendijk JA, Schilstra C. Statistical validation of normal tissue complication probability models. International journal of radiation oncology, biology, physics. 2012;84(1):e123–e9. 10.1016/j.ijrobp.2012.02.022 22541961

[15] Lee T-F, Chao P-J, Ting H-M, Chang L, Huang Y-J, Wu J-M, et al Using Multivariate Regression Model with Least Absolute Shrinkage and Selection Operator (LASSO) to Predict the Incidence of Xerostomia after Intensity-Modulated Radiotherapy for Head and Neck Cancer. PLoS ONE. 2014;9(2):1–11; e89700. 10.1371/journal.pone.0089700 PMC3938504 24586971

[16] Lee T-F, Huang E-Y. The Different Dose-Volume Effects of Normal Tissue Complication Probability Using LASSO for Acute Small-Bowel Toxicity during Radiotherapy in Gynecological Patients with or without Prior Abdominal Surgery. BioMed Res Inte. 2014;2014:1–9; 143020. 10.1155/2014/143020 PMC4124807 25136554

[17] Lee T-F, Liou M-H, Huang Y-J, Chao P-J, Ting H-M, Lee H-Y, et al LASSO NTCP predictors for the incidence of xerostomia in patients with head and neck squamous cell carcinoma and nasopharyngeal carcinoma. Sci Rep. 2014;4 10.1038/srep06217 PMC5385804 25163814

[18] Xu C-J, van der Schaaf A, Schilstra C, Langendijk JA, van’t Veld AA. Impact of statistical learning methods on the predictive power of multivariate normal tissue complication probability models. International journal of radiation oncology, biology, physics. 2012;82(4):e677–e84. 10.1016/j.ijrobp.2011.09.036 22245199

[19] Colombani C, Legarra A, Fritz S, Guillaume F, Croiseau P, Ducrocq V, et al Application of Bayesian least absolute shrinkage and selection operator (LASSO) and BayesCπ methods for genomic selection in French Holstein and Montbéliarde breeds. J Dairy Sci. 2012;96(1):575–91. 10.3168/jds.2011-5225 23127905

[20] Xu J, Yin J. Kernel least absolute shrinkage and selection operator regression classifier for pattern classification. Iet Comput Vis. 2013;7(1):48–55.

[21] Ramsey SD, Andersen MR, Etzioni R, Moinpour C, Peacock S, Potosky A, et al Quality of life in survivors of colorectal carcinoma. Cancer. 2000;88(6):1294–303. 10717609

[22] Stock JH, Watson MW. Generalized shrinkage methods for forecasting using many predictors. J Bus Econ Stat. 2012;30(4):481–93.

[23] Teppola P, Taavitsainen V-M. Parsimonious and robust multivariate calibration with rational function Least Absolute Shrinkage and Selection Operator and rational function Elastic Net. Anal Chim Acta. 2013;768(0):57–68. 23473250 10.1016/j.aca.2013.01.005

[24] Trotti A, Colevas AD, Setser A, Rusch V, Jaques D, Budach V, et al CTCAE v3.0: development of a comprehensive grading system for the adverse effects of cancer treatment. Semin Radiat Oncol. 2003;13(3):176–81. 12903007 10.1016/S1053-4296(03)00031-6

[25] Arriagada R, de Guevara J Cueto Ladron, Mouriesse H, Hanzen C, Couanet D, Ruffie P, et al Limited small cell lung cancer treated by combined radiotherapy and chemotherapy: evaluation of a grading system of lung fibrosis. Radiother Oncol. 1989;14(1):1–8. 2538863 10.1016/0167-8140(89)90002-9

[26] Lind PA, Svane G, Gagliardi G, Svensson C. Abnormalities by pulmonary regions studied with computer tomography following local or local-regional radiotherapy for breast cancer. International journal of radiation oncology, biology, physics. 1999;43(3):489–96. 10078627 10.1016/s0360-3016(98)00414-3

[27] Beetz I, Schilstra C, van der Schaaf A, van den Heuvel ER, Doornaert P, van Luijk P, et al NTCP models for patient-rated xerostomia and sticky saliva after treatment with intensity modulated radiotherapy for head and neck cancer: The role of dosimetric and clinical factors. Radiother Oncol. 2012;105(1):101–6. 10.1016/j.radonc.2012.03.004 22516776

[28] El Naqa I, Bradley J, Blanco AI, Lindsay PE, Vicic M, Hope A, et al Multivariable modeling of radiotherapy outcomes, including dose–volume and clinical factors. International journal of radiation oncology, biology, physics. 2006;64(4):1275–86. 16504765 10.1016/j.ijrobp.2005.11.022

[29] Tibshirani R. Regression shrinkage and selection via the lasso. J Roy Stat Soc B. 1996:267–88.

[30] Hassaballa HA, Cohen ES, Khan AJ, Ali A, Bonomi P, Rubin DB. Positron emission tomography demonstrates radiation-induced changes to nonirradiated lungs in lung cancer patients treated with radiation and chemotherapy. Chest. 2005;128(3):1448–52. 10.1378/chest.128.3.1448 . 16162742

[31] Choi YW, Munden RF, Erasmus JJ, Park KJ, Chung WK, Jeon SC, et al Effects of radiation therapy on the lung: radiologic appearances and differential diagnosis. Radiographics: a review publication of the Radiological Society of North America, Inc. 2004;24(4):985–97; discussion 98. 10.1148/rg.244035160 . 15256622

[32] Ikezoe J, Takashima S, Morimoto S, Kadowaki K, Takeuchi N, Yamamoto T, et al CT appearance of acute radiation-induced injury in the lung. AJR American journal of roentgenology. 1988;150(4):765–70. 10.2214/ajr.150.4.765 . 3258086

[33] Beetz I, Schilstra C, van Luijk P, Christianen ME, Doornaert P, Bijl HP, et al External validation of three dimensional conformal radiotherapy based NTCP models for patient-rated xerostomia and sticky saliva among patients treated with intensity modulated radiotherapy. Radiother Oncol. 2012;105(1):94–100. 10.1016/j.radonc.2011.11.006 22169766

[34] Goldman UB, Wennberg B, Svane G, Bylund H, Lind P. Reduction of radiation pneumonitis by V 20-constraints in breast cancer. Radiat Oncol. 2010;5(99):1–6. 21034456 10.1186/1748-717X-5-99 PMC2987943

[35] Kahán Z, Csenki M, Varga Z, Szil E, Cserháti A, Balogh A, et al The risk of early and late lung sequelae after conformal radiotherapy in breast cancer patients. International journal of radiation oncology, biology, physics. 2007;68(3):673–81. 17350177 10.1016/j.ijrobp.2006.12.016

[36] Wennberg B, Gagliardi G, Sundbom L, Svane G, Lind P. Early response of lung in breast cancer irradiation: radiologic density changes measured by CT and symptomatic radiation pneumonitis. International journal of radiation oncology, biology, physics. 2002;52(5):1196–206. 11955730 10.1016/s0360-3016(01)02770-5

[37] Gagliardi G, Bjöhle J, Lax I, Ottolenghi A, Eriksson F, Liedberg A, et al Radiation pneumonitis after breast cancer irradiation: analysis of the complication probability using the relative seriality model. International journal of radiation oncology, biology, physics. 2000;46(2):373–81. 10661344 10.1016/s0360-3016(99)00420-4

[38] Theuws J, Kwa S, Wagenaar A, Boersma L, Damen E, Muller S, et al Dose–effect relations for early local pulmonary injury after irradiation for malignant lymphoma and breast cancer. Radiother Oncol. 1998;48(1):33–43. 9756170 10.1016/s0167-8140(98)00019-x

[39] Abe M, Miwa H, Ema R, Miki Y, Tomita K, Nakamura H, et al Risk Factors Of Symptomatic Radiation Pneumonitis In Post-Operative Breast Cancer Patients. Am J Resp Crit Care. 2012;185:A5727.

[40] Allen AM, Prosnitz RG, Ten Haken RK, Normolle DP, Yu X, Zhou S-m, et al Body mass index predicts the incidence of radiation pneumonitis in breast cancer patients. The Cancer Journal. 2005;11(5):390–8. 16267908 10.1097/00130404-200509000-00006

[41] Fu W, Dai J, Hu Y. The influence of lateral electronic disequilibrium on the radiation treatment planning for lung cancer irradiation. Bio-Med Mater Eng. 2004;14(1):123–6. 14757959

[42] Hardman P, Tweeddale P, Kerr G, Anderson E, Rodger A. The effect of pulmonary function of local and loco-regional irradiation for breast cancer. Radiother Oncol. 1994;30(1):33–42. 8153378 10.1016/0167-8140(94)90007-8

[43] Lind P, Bylund H, Wennberg B, Svensson C, Svane G. Abnormalities on chest radiographs following radiation therapy for breast cancer. Eur Radiol. 2000;10(3):484–9. 10757001 10.1007/s003300050081

[44] Taghian AG, Assaad SI, Niemierko A, Kuter I, Younger J, Schoenthaler R, et al Risk of pneumonitis in breast cancer patients treated with radiation therapy and combination chemotherapy with paclitaxel. J Natl Cancer I. 2001;93(23):1806–11. 10.1093/jnci/93.23.1806 11734597

[45] Hooning MJ, Aleman BM, van Rosmalen AJ, Kuenen MA, Klijn JG, van Leeuwen FE. Cause-specific mortality in long-term survivors of breast cancer: a 25-year follow-up study. International journal of radiation oncology, biology, physics. 2006;64(4):1081–91. 16446057 10.1016/j.ijrobp.2005.10.022

[46] Johansson S, Bjermer L, Franzen L, Henriksson R. Effects of ongoing smoking on the development of radiation-induced pneumonitis in breast cancer and oesophagus cancer patients. Radiother Oncol. 1998;49(1):41–7. 9886696 10.1016/s0167-8140(98)00064-4

